# Magnetization due to localized states on graphene grain boundary

**DOI:** 10.1038/srep11744

**Published:** 2015-07-06

**Authors:** Sudipta Dutta, Katsunori Wakabayashi

**Affiliations:** 1 WPI-International Center for Materials Nanoarchitechtonics (WPI-MANA), National Institute for Materials Science (NIMS), Namiki 1-1, Tsukuba, Ibaraki – 3050044, Japan; 2 International Center for Young Scientists (ICYS), WPI-International Center for Materials Nanoarchitechtonics (WPI-MANA), National Institute for Materials Science (NIMS), Namiki 1-1, Tsukuba, Ibaraki – 3050044, Japan; 3 Department of Nanotechnology for Sustainable Energy, School of Science and Technology, Kwansei Gakuin University, Gakuen 2-1, Sanda 669-1337, Japan

## Abstract

Magnetism in graphene has been found to originate from various defects, e.g., vacancy, edge formation, add-atoms etc. Here, we discuss about an alternate route of achieving magnetism in graphene via grain boundary. During chemical vapor deposition of graphene, several graphene nucleation centers grow independently and face themselves with unusual bonding environment, giving rise to the formation of grain boundaries. We investigate the origin of magnetism in such grain boundaries within first-principles calculations, by letting two nucleation centers interact with each other at their interface. We observe formation of unprecedented point defect, consisting of fused three-membered and larger carbon rings, which induces net magnetization to graphene quantum dots. In case of periodic lattices, the appearance of array of point defects leads to the formation of magnetic grain boundaries. The net magnetization on these defects arises due to the deviation from bipartite characteristics of pristine graphene. We observe magnetic grain boundary induced dispersion less flat bands near Fermi energy, showing higher localization of electrons. These flat bands can be accessed via small doping, leading to enhanced magnetism. Moreover, the grain boundaries can induce asymmetric spin conduction behavior along the cross boundary direction. These properties can be exploited for sensor and spin-filtering applications.

Introduction of magnetism in graphene in terms of defects has been gaining tremendous impetus in recent times, owing to their application possibilities towards spin transport and sensor devices^1^,^2^,^3^,^4^,^5^,^6^,^7^,^8^,^9^. The basic idea of this magnetism lies in Lieb’s theorem for bipartite lattice^10^, which says: the inequality between two sublattice points, A and B, i.e., N_A_ ≠ N_B_ introduces net magnetic moment. Being a bipartite lattice, this theorem is valid in case of graphene as well. The graphene unitcell consists of two sublattice points, A and B that prefer opposite spin occupancies, making the over all net magnetization zero (see Fig. 1a). Therefore, it is expected that, the vacancy defects can introduce inequality between N_A_ and N_B_, making the two-dimensional graphene magnetic^11^,^12^,^13^,^14^,^15^,^16^,^17^,^18^. However, the formation of zigzag edges, a defect, introduced by finite termination of graphene along a certain crystallographic direction can also introduce net magnetization, in spite of N_A_ = N_B_^19^,^20^,^21^,^22^. This magnetization in semi-infinite zigzag edge graphene can be attributed to the formation of peculiar edge localized states near Fermi energy and the spins tend to align parallelly on the same sublattice points along the same edge, giving rise to a long-range ferrimagnetic coupling^23^,^24^,^25^. However, formation of another parallel zigzag edge in a ribbon like geometry results in total zero magnetization by putting the spins in opposite polarization on the other sublattice points along the opposite edge. In spite of such antiferromagnetic alignment of spins between two opposite edges, the local spin moments along the edges remain robust. These edge localized states have been studied within different theoretical formalisms and very recently, they have been confirmed experimentally within scanning tunneling spectroscopy^26^. Moreover, introduction of holes can induce net magnetization in zigzag edge graphene nanoribbons^27^,^28^,^29^. In this paper, we will show another origin of the magnetism in graphene, i.e., unusual bonding and hybridization. This is owing to the catenation property of carbon, which allows diverse hybridization possibilities, especially during the chemical vapor deposition (CVD) growth of graphene.

Although, there exist few experimental techniques, like mechanical exfoliation^30^,^31^,^32^,^33^ or graphene oxide reduction^34^,^35^ for preparation of graphene, the CVD technique has been proved to be the most efficient way of preparing transferrable large area graphene samples^36^,^37^,^38^,^39^,^40^,^41^,^42^,^43^,^44^,^45^,^46^. Moreover, the CVD grown graphene has recently been demonstrated for transparent and flexible device applications^37^, indicating a huge technological leap. However, the growth of wafer scale graphene within this technique often results in several kinds of defects like point defects (PDs) or grain boundaries (GBs) among pristine graphene domains with different crystallographic orientations^47^,^48^,^49^,^50^,^51^,^52^. These PDs and GBs can significantly modify the electronic and transport properties of graphene samples. Therefore, the knowledge of these defects is essential for the realization of their applications. Moreover, the bipartite characteristics may collapse over these defects (see Fig. 1b), giving rise to unusual magnetic properties. In this paper, we have theoretically simulated the formation of several PDs and GBs between two pristine graphene domains during CVD growth and have shown the appearance of defect-induced magnetism.

During diffusive growth of graphene on some substrate, there is formation of several nucleation centers that grow independently until they merge finally at their interface with smooth or mismatched crystallographic angle^47^,^50^,^51^. To simulate the CVD growth of graphene computationally, we start with a minimal approximation. We first consider two small segments of graphene (as shown in Fig. 2a), as if they are part of two nucleation centers and let them interact with each other. Note that, the valency of the sp^2^ hybridized carbon atoms along the edges is passivated with hydrogen, except in the shaded region. This approximation ensures that, the graphene segments are part of big single crystalline graphene domains with all satisfied valencies and they can only interact within themselves along their interface region (shaded region) with active carbon atoms of unsatisfied valencies. Now, the structural relaxation results in merging of two domains with or without defects. Here defect refers to the formation of non-hexagonal carbon rings. Since, we consider quantum dot like small segments in Fig. 2a for computational ease, the defects should be considered as point defects (PDs), due to their non-periodic nature. However, we shall bring in the discussions about the formation of grain boundaries (GBs) in between periodic graphene domains in later sections.

Note that, we have not considered the underlying substrate explicitly in our calculations. However, we believe that, the simulation technique of surface diffusion of carbon atoms as adopted here can mimic the CVD growth of graphene on Cu surface^45^. Very low solubility of carbon atoms in Cu even at very high temperature restricts the diffusion of carbon atoms in the bulk of metal substrate as observed in case of Ni or Co substrates. Consequently, during the CVD growth, carbon atoms diffuse on Cu substrate surface to produce mainly single layer graphene^45^. Moreover, in our calculations, the faces of the CVD grown graphene domains have been considered to be zigzag as observed experimentally and predicted by theoretical calculations^53^,^54^,^55^. In addition to that, the growth of graphene on Cu(111) plane results in highly uniform orientation of hexagons due to the symmetry match between graphene and the underlying substrate^53^,^56^, as has been considered in the present study. However, the structural and electronic aspects of the grain boundaries within these oriented graphene domains are still not explored satisfactorily.

## Results

**Figure 2:**
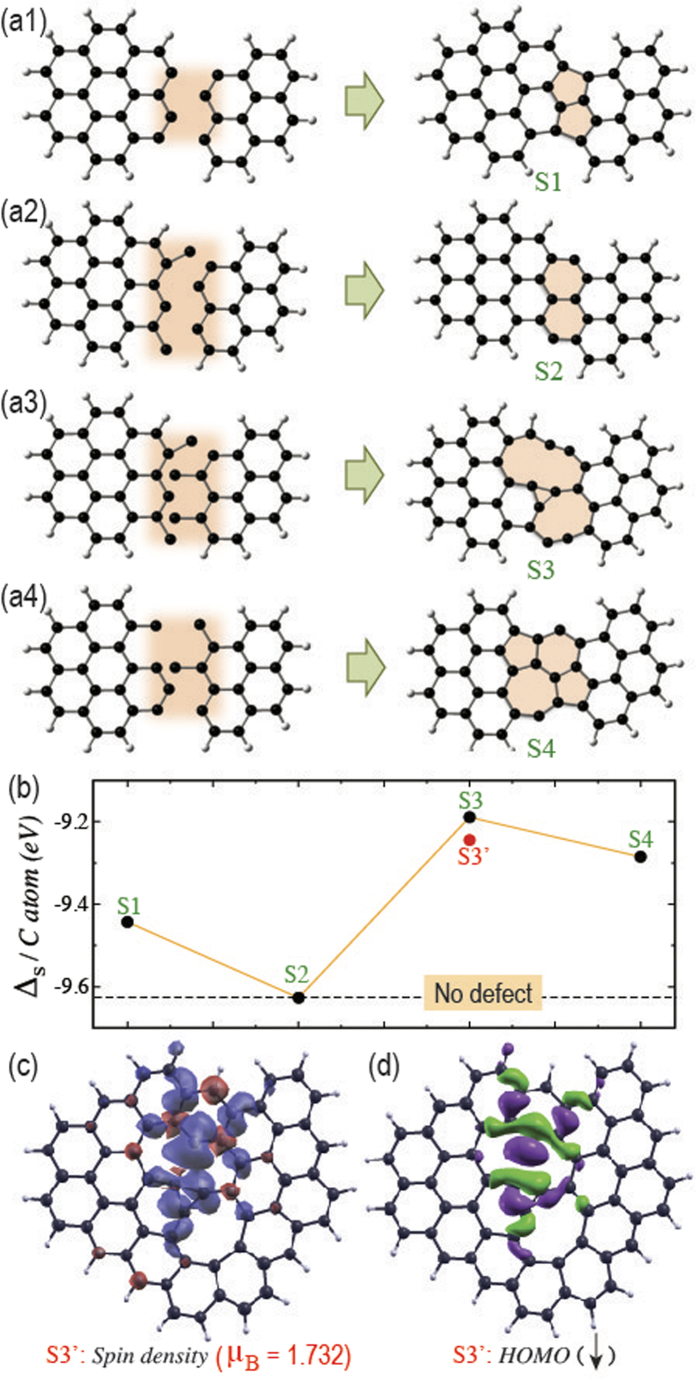
Appearance of net magnetization on point defects. The initial structures (left) before geometrical relaxation with unpassivated carbon atoms of two small graphene segments interacting at the interface (shaded region) and the corresponding optimized structures (right) after geometrical relaxation, namely S1, S2, S3 and S4 with the formation of point defects (shaded region) are shown in (**a1**), (**a2**), (**a3**) and (**a4**), respectively. (**b**) The stabilization energy per carbon atom (Δ_S_/C atom) for the above mentioned systems are shown along with that of the system, S3’ (shown in Fig. 2c). The dashed line indicates the Δ_S_/C atom for the system without any defect, i.e., S2 after passivating the dangling bonds at the shaded region by hydrogen atom. (**c**) The spin density plot for S3’, showing localization of spins over the defect, giving rise to net magnetization of 1.732 Bohr magneton in the system. Two colors indicate densities for two different spin polarizations. (**d**) The wave function of the highest occupied molecular orbital (HOMO) for the majority spin (here down spin) is localized over the defect.

We perform structural relaxations with several starting geometries varying the position and number of carbon atoms at the interface of two quantum dot like graphene segments and present four representative structures, namely S1, S2, S3 and S4. Our calculations always result in planar structures with varying geometry and bonding at the interface, as depicted by the shaded region in Fig. 2a1–a4. In case of S2, the two graphene segments merge smoothly with formation of hexagonal carbon rings at the interface. In all other cases, we observe formation of PDs with non-hexagonal carbon rings. That is why; we analyze the stability of the PDs in comparison with the defect free S2 structure in Fig 2b. Here we plot, the stabilization energy per carbon atom (Δ_S_ / C atom) for all the relaxed structures, which have been calculated by the following equation,


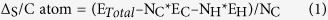


where, E_*Total*_, E_C_ and E_H_ are the energies of the relaxed structures (S1, S2, S3 and S4), one carbon atom and one hydrogen atom, respectively and N_C_ N_H_ is the number of carbon (hydrogen) atoms in the system. Note that, the stabilization energy of S2 as shown in Fig. 2b is calculated after passivating the dangling bonds of two edge carbon atoms at the interface (shaded region). This is to make sure that, the benchmark geometry S2 really reflects the defect free energy. As can be seen, the PDs in S1 and S4 show the formation of five and seven membered carbon rings, which have been observed experimentally in the existing literature. However, the observation of three membered ring surrounded by larger rings with 8–10 carbon atoms in S3 is unprecedented. We observe the formation of such PDs in several other geometrical relaxations also. As we know, the stability of isolated three membered carbon ring is less compared to the hexagonal rings, owing to the lack of aromatic stability. Therefore, the lesser stability of S3 is expected (see Fig. 2b). However, the presence of larger rings around the three membered ring provides some stability to this kind of PDs. To check the stability of such PD in larger graphene domains, we keep adding carbon atoms surrounding the PD and subsequently relax the whole structures. We observe that, the PD remains unchanged even in larger domains with little deformation of the larger rings (see Fig. 2c). Moreover, the stabilization energy increases with increase in the quantum dot size as can be seen for S3’. These observations indicate that, the formation of such PDs in large graphene domain is possible. Although, the thermodynamic stabilities of such PDs are less compared to the defect free graphene, they can be formed with kinetic stability during the CVD growth. This is due to the fact that, the lateral movements or rotations of large graphene nucleations are arrested due to their larger size and may result in undesired defects at the interface with unusual bonding characteristics.

Now it is obvious that, the bipartite nature of pristine graphene collapses at such defects and discrimination of N_A_ and N_B_ becomes impossible. That may lead to asymmetric distribution of up and down spins and may induce net magnetization. Although, the appearance of magnetization is not always obvious as observed in case of Stone-Well’s defect, i.e., the fused 5 and 7 membered rings, which does not prefer any local spin moments^57^,^58^,^59^,^60^,^61^. However, our calculations in case of S3’ clearly show that, one kind of spin is preferred over the PD (see Fig. 2c), giving rise to a net magnetization of 1.732 Bohr magneton. Note that, S3’ contains even number of carbon atoms (60 C atoms). So the magnetization cannot appear due to the presence of unpaired spins, which can arise in case of systems with odd number of carbon atoms with N_A_ ≠ N_B_. Rather, it results due to the collapse of the bipartite nature over the PD. Moreover, the observed magnetization does not appear only due to the presence of zigzag edges. Although, the appearance of local spin moments at zigzag edges has been observed in S3’, their contribution is far too less compared to the local spin moment over the PD and cannot be seen within the considered isovalue in Fig. 2c. As can be seen, the majority spin moment is mainly localized on the valency-unsatisfied carbon atom in the ten-membered ring and on the three-membered ring (see Fig. 2c). The wave-function analysis shows that, appearance of such magnetization can be attributed to the localized highest occupied molecular orbital (HOMO) of the majority spin (down spin in present case) over the PD (see Fig. 2d). All other wave functions near the Fermi energy are delocalized over the full structure of S3’.

The appearance of localized states in case of zigzag edge graphene nanoribbons has been explored in great extent in the existing literature and observed experimentally as well^19^,^20^,^23^,^26^,^62^. After the observation of localized wave-function with net magnetization over PDs, we asked the question: is it possible to obtain the array of such magnetic PDs, in other word, magnetic GBs which can result in localized states in the bulk of graphene? Because such localized magnetic states in the proximity of Fermi energy can be exploited greatly for spin-transport devices.

**Figure 3:**
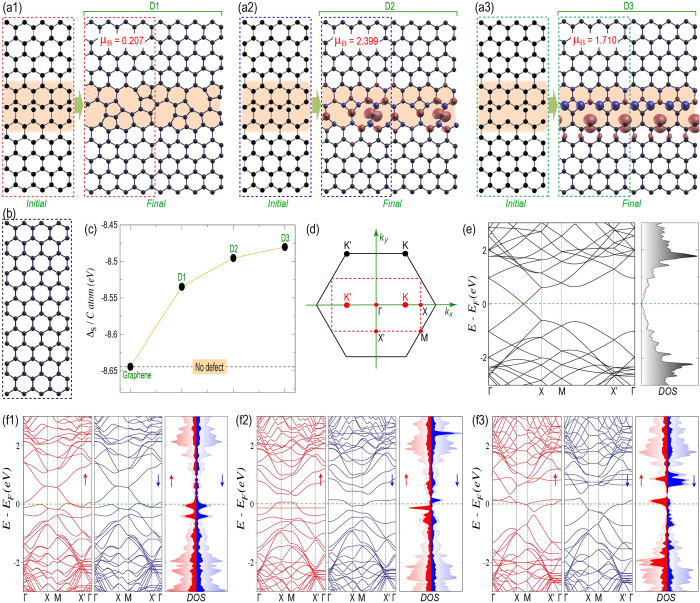
Grain boundary induced magnetization in two-dimensional graphene. The initial structures before geometrical relaxation with two graphene grains in the supercell (dashed box) interacting at their interface (shaded region) and the corresponding optimized final structures, namely D1, D2 and D3 with the formation of grain boundaries (shaded region) are shown along with their spin density and net magnetization of 0.207, 2.399 and 1.710 Bohr magneton per supercell in (**a1**), (**a2**) and (**a3**), respectively. (**b**) The rectangular graphene supercell of size comparable with that of D1, D2 and D3. (**c**) The stabilization energy per carbon atom (Δ_S_/C atom) for D1, D2 and D3 along with that of pristine graphene (dashed line). (**d**) The Brillouin zone of graphene for rhombus unit cell (solid hexagon) and that for rectangular supercell (dashed rectangle) with the high symmetric points. (**e**) The band structure and DOS of rectangular graphene supercell. (**f1**), (**f2**) and (**f3**) represent the spin polarized band structures and DOS for up and down spins in case of D1, D2 and D3, respectively. The total DOS and pDOS from the grain boundaries are shown by light and dark shades, respectively. The horizontal dashed lines in band structures and density of states indicate the position of Fermi energy, while the vertical dashed lines show the location of high symmetric points.

That is why, we next consider the periodic supercells with two graphene grains facing each other with zigzag and Klein edges, as can be seen in Fig. 3a1. These two grains with zero crystallographic angle mismatch, that can be obtained experimentally via highly oriented graphene growth on Cu(111) surface^53^,^54^,^55^,^56^, interact with each other at their interface as depicted by the shaded region. Upon structural relaxation, they form a grain boundary along the interface with fused five and eight membered rings. This GB is non-magnetic in nature, as evident from its negligible net magnetization and no spin polarization. Then we start modifying the interface, as we have done during the investigation of PDs in previous section, and relax the whole supercell. We present two such GBs in D2 (Fig. 3a2) and D3 (Fig. 3a3). Both these GBs are magnetic in nature with considerable net magnetization per supercell of 2.399 and 1.710 Bohr magneton, respectively. The wave functions are localized over the GBs as evident from the spin density plots (see Fig. 3a2,a3). Although, the supercell in D2 contains odd number of carbon atoms, the number of unpaired spins is more than one. That clearly indicates that, the net magnetization is not solely due to N_A_ ≠ N_B_. Moreover, in spite of even number of carbon atoms in the supercell of D3, the appearance of localized spin moments over the GB proves our argument of unusual bonding induced magnetization. Note that, these magnetic GBs contain four-eight and four-ten membered fused rings, respectively.

All these GBs are less stable compared to the pristine graphene (in Fig. 3b), as can be seen from Fig. 3c. However, these GBs can be formed with kinetic stability during the CVD growth, as we have argued before. Note that, all these GBs maintain the planarity of the whole system, showing the strength of sp^2^ network. Now, before entering the electronic properties of these GBs, we first discuss about the modified Brillouin zone in Fig. 3d. As can be seen, the hexagonal Brillouin zone (solid line) for rhombus unit cell of graphene is now mapped to a rectangular Brillouin zone (dashed line) in case of rectangular graphene supercell, as shown in Fig. 3b. Due to the zone folding in the modified supercell, the Dirac points (K and K’) are now positioned over the line connecting two high symmetric points Γ and X. The Dirac linear dispersion can now be observed at the K point in between Γ and X (see Fig. 3e).

However, in case of GB formation in D1, D2 and D3, the linear dispersion completely disappears with appearance of metallic density of states (DOS) at Fermi energy, as can be seen from Fig. 3f1,3f2 and f3, respectively. The projected DOS (pDOS) shows that the states at / near Fermi energy mainly arise from the GBs. In case of D1, the degeneracy between up and down spin bands (see Fig. 3f1) can be attributed to the absence of any spin preference over the grain boundary. This behavior is analogous to the periodic Stone-Wales defect that does not allow any spin preference and shows metallic behavior with degenerate up and down spin bands^57^,^58^,^59^,^60^,^61^. However, in case of D2 and D3, the net magnetization and spin polarization over the GBs result in asymmetric up and down spin bands (see Fig. 3f2,f3). As can be seen, in case of D2, a completely dispersion less flat band for up spin appears just below the Fermi energy (−0.15 eV). However, for down spin, the almost dispersion less band appears above the Fermi energy (0.14 eV). These dispersion less bands indicate that the electrons are highly localized and do not carry any velocity. Therefore, the GB in D2 would act as scattering barrier for the cross GB transport. Moreover, it is obvious that, in case of such GB, the system will show higher net magnetization upon small electron or hole doping when the Fermi energy gets aligned with the observed flat bands above or below the Fermi energy, respectively. On the contrary, in case of D3, the bands for both the spins crossing the Fermi energy are dispersive and therefore the electrons are conducting. Note that, for cross GB transport the system needs dispersive bands along X – M and Γ - X' directions. As can be seen in Fig. 3f3, both the spin components have dispersive bands along Γ - X'. However, the dispersive band along X – M exists only for the up spin component. This asymmetry between up and down spins is reflected in their spin polarized DOS as well. Therefore, one can expect asymmetric spin transport across the GB and consequent spin-filtering behavior.

**Figure 4:**
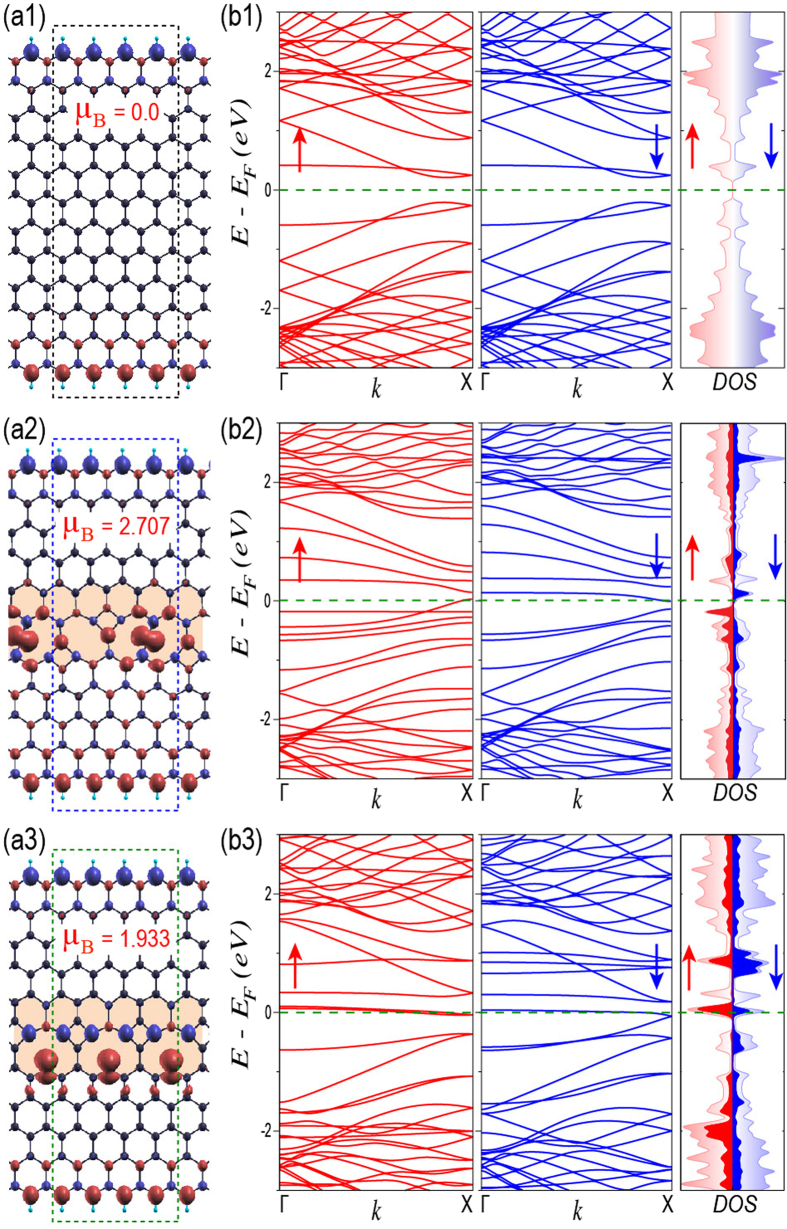
Magnetic grain boundaries in zigzag edge graphene nanoribbons. The optimized geometries of zigzag edge graphene nanoribbon and that of with grain boundaries (as in Fig. 3a2,a3), marked by shaded region are shown in (**a1**), (**a2**) and (**a3**), respectively along with their spin density and net magnetization of 0.0, 2.707 and 1.933 Bohr magneton per supercell. The dashed boxes indicate the supercell. (**b1**), (**b2**) and **(b3**) show the spin polarized band structures and DOS for up and down spins in case of systems, shown in (**a1**), (**a2**) and (**a3**), respectively. The total DOS and pDOS from the grain boundaries are shown by light and dark shades, respectively. The horizontal dashed lines in band structures and density of states indicate the position of Fermi energy.

Furthermore, we explore the behavior of these magnetic GBs in presence of graphene edges. Before discussing about their electronic properties, we first present the much-studied zigzag graphene nanoribbon system in absence of any defect and show its spin density and electronic properties in Fig. 4a1,b1, respectively. As we know, the spins of opposite polarization localize on either edges to make the overall zero net magnetization (see Fig. 4a1)^24^,^25^,^63^. In addition to this, the partial flat bands, i.e., the edge states at Fermi energy, as obtained from tight-binding calculations, split with the incorporation of electronic correlations, resulting in semiconducting band gap at Fermi energy (see Fig. 4b1)^27^,^28^,^63^.

When we create similar interfacial environment (as in D2 and D3) in case of ribbons, we obtain identical GBs after geometrical relaxation, as can be seen in Fig. 4a2,a3. However, the net magnetization of the total supercell is enhanced as compared to their two-dimensional counterparts. This occurs due to the coupling of the GBs with the spin polarized edges of the ribbons. We believe that, for sufficiently wider ribbons, the GBs at the bulk will be completely decoupled with the edges and the net magnetization will become identical to the two-dimensional systems. The spin density plots show the localization of non-bonding electrons with abundance of one kind of spin over the GBs. This spin localization is analogous to that of the edges. However, the absence of its counterpart with opposite spin localization, unlike two edges clearly makes these GBs advantageous over the defect less ribbons in view of their unexplored electronic properties. The presence of GBs introduces dispersion less flat bands at/near Fermi energy, while the edge states still remain away from the Fermi energy (see Fig. 4b2,b3), as evident from the DOS and pDOS plots. In case of first magnetic GB, the flat bands of two different spin polarizations are positioned in either sides of the Fermi energy, indicating the magnetic excitations upon electron or hole doping. Whereas, in case of second magnetic GB, the flat bands for both the spins are aligned with the Fermi energy, showing higher localization of non-bonding electrons and absence of any conduction spins along the GB. However, it is worth exploring the transport probabilities across the GB and will be addressed elsewhere.

## Discussion

In conclusion, we have observed the formation of magnetic point defects and grain boundaries during the CVD growth of graphene within ab-initio framework. The simulation procedure of defect formation via allowing two graphene segments to interact with each other, as adopted in this study is simple but unprecedented. Moreover, for the first time we detect the possibility of a point defect of three-membered carbon ring with surrounding larger rings that can induce net magnetization to the graphene sample through the spin polarized localized states. The observation of stable three-membered carbon ring is interesting from fundamental chemistry viewpoint and can be detected via scanning tunneling microscope (STM) experiment due to higher charge localization. In case of periodic systems, however the magnetic GBs are built by the fusion of four membered rings with larger carbon rings. These observations lead to the conclusion that, the existence of smaller rings in the defect indulges the formation of larger rings which localize the non-bonding electrons to give rise to net magnetization. As a whole, this phenomenon arises due to the deviation from the graphene bipartite nature at the defect. The electronic properties show the possibilities of doping induced magnetic excitations and asymmetric spin transports across the GBs. Therefore, these unprecedented magnetic defects can be useful for sensor and spin-filtering applications. Note that, the geometrical aspects of PDs and GBs can be completely stochastic depending on the interfacial environment within graphene grains during CVD growth. But, our argument that, the formation of localized states will always give rise to magnetism, remains generalized. There exist a vast room for exploration of these defects with potential application possibilities. Our present study is just the first step towards the understanding of the GB induced magnetism in a purely carbon system.

## Methods

For the structural relaxation of CVD grown graphene and for the investigation of the electronic properties of the same in presence of PDs and GBs, we adopt *ab-initio* level of calculations as implemented in SIESTA^64^. Generalized gradient approximation (GGA) has been considered within Perdew-Burke-Ernzerhof (PBE) exchange and correlation functional^65^. Spin polarized calculations have been performed with double zeta polarized (DZP) basis set and energy cut-off of 400 Ry for real space mesh size. Note that, we consider initial antiferromagnetic spin orientation guess for the wave-function as evident in case of graphene. All the structures have been relaxed along with the relaxation of lattice constants of supercells until the force on each atom reaches 0.04 eV/Å and sufficient vacuum has been created along the non-periodic directions to avoid any interactions within adjacent supercells. We consider Brillouin zone sampling over 10 × 10 × 1 and 70 × 1 × 1 Monkhorst-Pack grid for two-dimensional graphene (Fig. 3) and for quasi-one-dimensional graphene nanoribbons (Fig. 4), respectively. However for non-periodic quantum dot structures (as shown in Fig. 2), we perform only Γ-point calculations.

## Additional Information

**How to cite this article**: Dutta, S. and Wakabayashi, K. Magnetization due to localized states on graphene grain boundary. *Sci. Rep.* **5**, 11744; doi: 10.1038/srep11744 (2015).

## Figures and Tables

**Figure 1:**
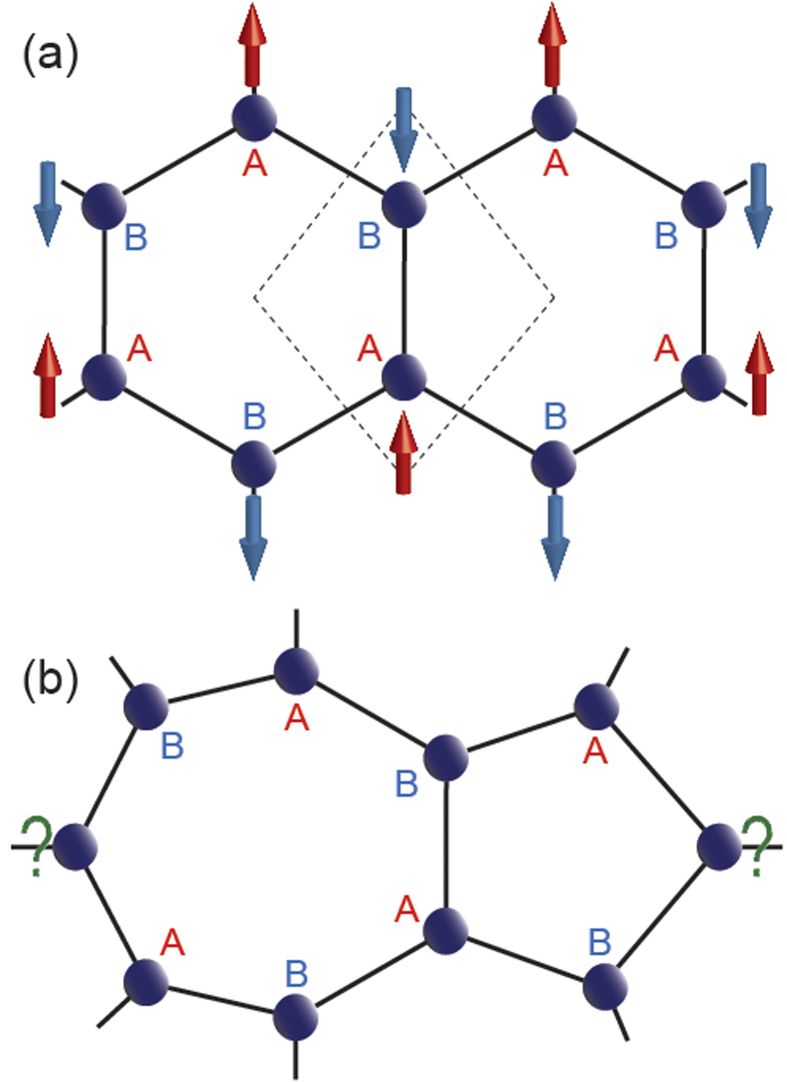
Schematic representation of Lieb’s theorem on bipartite lattice. (**a**) The graphene lattice with rhombus unit cell (dashed box) consisting of two distinct sublattice points, A and B that prefer to localize opposite spins, making the whole system antiferromagnetic with same number of A and B sublattice points, i.e., N_A_ = N_B_. Note that, each A (B) sublattice point is connected to three B (A) sublattice points. (**b**) One commonly known defect in graphene, namely Stone-Well’s defect consisting of fused five and seven membered rings. The bipartite characteristic collapses in such defects due to undefined sublattice nature emphasized by the “?” marks, giving rise to unusual magnetic properties.
